# Spike-Timing-Dependent Plasticity Mediated by Dopamine and its Role in Parkinson’s Disease Pathophysiology

**DOI:** 10.3389/fnetp.2022.817524

**Published:** 2022-03-04

**Authors:** Mojtaba Madadi Asl, Abdol-Hossein Vahabie, Alireza Valizadeh, Peter A. Tass

**Affiliations:** ^1^ Department of Physics, Institute for Advanced Studies in Basic Sciences (IASBS), Zanjan, Iran; ^2^ School of Electrical and Computer Engineering, College of Engineering, University of Tehran, Tehran, Iran; ^3^ Department of Psychology, Faculty of Psychology and Education, University of Tehran, Tehran, Iran; ^4^ Department of Neurosurgery, Stanford University School of Medicine, Stanford, CA, United States

**Keywords:** spike-timing-dependent plasticity, dopamine, Parkinson’s disease, beta oscillations, basal ganglia

## Abstract

Parkinson’s disease (PD) is a multi-systemic neurodegenerative brain disorder. Motor symptoms of PD are linked to the significant dopamine (DA) loss in substantia nigra pars compacta (SNc) followed by basal ganglia (BG) circuit dysfunction. Increasing experimental and computational evidence indicates that (synaptic) plasticity plays a key role in the emergence of PD-related pathological changes following DA loss. Spike-timing-dependent plasticity (STDP) mediated by DA provides a mechanistic model for synaptic plasticity to modify synaptic connections within the BG according to the neuronal activity. To shed light on how DA-mediated STDP can shape neuronal activity and synaptic connectivity in the PD condition, we reviewed experimental and computational findings addressing the modulatory effect of DA on STDP as well as other plasticity mechanisms and discussed their potential role in PD pathophysiology and related network dynamics and connectivity. In particular, reshaping of STDP profiles together with other plasticity-mediated processes following DA loss may abnormally modify synaptic connections in competing pathways of the BG. The cascade of plasticity-induced maladaptive or compensatory changes can impair the excitation-inhibition balance towards the BG output nuclei, leading to the emergence of pathological activity-connectivity patterns in PD. Pre-clinical, clinical as well as computational studies reviewed here provide an understanding of the impact of synaptic plasticity and other plasticity mechanisms on PD pathophysiology, especially PD-related network activity and connectivity, after DA loss. This review may provide further insights into the abnormal structure-function relationship within the BG contributing to the emergence of pathological states in PD. Specifically, this review is intended to provide detailed information for the development of computational network models for PD, serving as testbeds for the development and optimization of invasive and non-invasive brain stimulation techniques. Computationally derived hypotheses may accelerate the development of therapeutic stimulation techniques and potentially reduce the number of related animal experiments.

## 1 Introduction

Spike-timing-dependent plasticity (STDP) is a fundamental mechanism in the brain that modifies the synaptic strengths between neurons based on the coincidence of pre- and postsynaptic spikes (Gerstner et al., 1996; Markram et al., 1997; Bi and Poo, 1998). In conventional asymmetric forms of STDP (Bi and Poo, 1998), the temporal order of spikes is critical, so that when the presynaptic spike precedes the postsynaptic spike (i.e., pre-post pairing), the STDP rule leads to long-term potentiation (LTP) of the synapse between pre- and postsynaptic neurons, whereas long-term depression (LTD) is induced in the reverse scenario (i.e., post-pre pairing) (Markram et al., 1997). Although STDP is a local mechanism and merely depends on the pre- and postsynaptic spike timings (Bi and Poo, 1998), it can determine global connectivity patterns emerging in recurrent neuronal networks (Song and Abbott, 2001; Izhikevich et al., 2004; Lubenov and Siapas, 2008; Gilson et al., 2009; Kozloski and Cecchi, 2010; Madadi Asl et al., 2017; Madadi Asl et al., 2018b). In this way, neuronal activity shapes synaptic connectivity through STDP, which in turn, determines the activity pattern of neurons in the network (Gilson et al., 2010; Kozloski and Cecchi, 2010; Knoblauch et al., 2012). The interplay between neuronal activity and synaptic connectivity ultimately determines the emerging structure and dynamics of the network (Aoki and Aoyagi, 2009; Madadi Asl et al., 2018c; Ziaeemehr et al., 2020; Ziaeemehr and Valizadeh, 2021).

Regulation of the synaptic strengths due to the time difference between pre- and postsynaptic spikes in the context of STDP was experimentally observed in cortical (Markram et al., 1997; Sjöström et al., 2001; Froemke and Dan, 2002) and hippocampal (Magee and Johnston, 1997; Bi and Poo, 1998) slices about 2 decades ago. Later, several experimental studies reported evidence of STDP in different pathways of the basal ganglia (BG) (see (Wickens, 2009) for an overview). In particular, numerous studies were devoted to synaptic plasticity observed in cortico-striatal connections (Mahon et al., 2004; Fino et al., 2005; Fino et al., 2008; Kreitzer and Malenka, 2008; Di Filippo et al., 2009; Fino et al., 2010) (reviewed by (Fino and Venance, 2010)). The striatum provides the main input to the BG and is massively subjected to activity-dependent long-term synaptic changes (Charpier and Deniau, 1997). These long-term changes of the synaptic strengths are believed to play a key role in adaptive motor control (Graybiel et al., 1994) and learning processes (Packard and Knowlton, 2002; Di Filippo et al., 2009). Long-term modification of synapses was also observed in other parts of the BG, e.g., within rat subthalamic nucleus (STN) neurons induced by high-frequency stimulation (HFS) (Shen et al., 2003).

**FIGURE 1:**
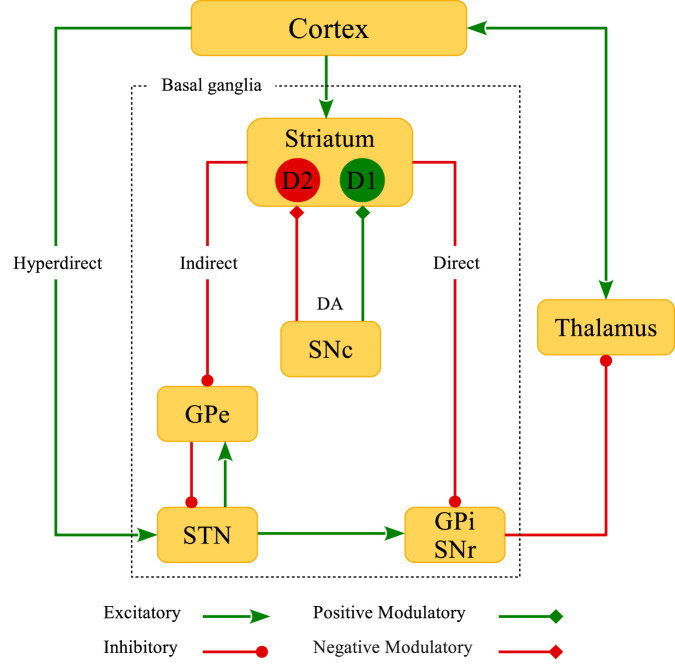
Schematic representation of the classical model of BG circuitry. BG consist of several subcortical nuclei (inside the dotted rectangle): The striatum, external (GPe) and internal (GPi) segments of the globus pallidus, substantia nigra pars compacta (SNc) and pars reticulata (SNr), and the subthalamic nucleus (STN). Direct, indirect, hyperdirect and DA D1R- and D2R-mediated pathways are also shown in the figure.

The BG are the main subcortical nuclei characterized by four primary parts (shown in Figure 1): 1) The striatum is divided into ventral and dorsal segments. The ventral striatum consists of the nucleus accumbens (NAc) and the olfactory tubercle. The dorsal striatum consists of the caudate nucleus and the putamen. The caudate is associated with limbic functions, whereas the putamen is related to the motor functions of the striatum, as implicated in Parkinson’s disease (PD) pathophysiology (Threlfell and Cragg, 2011; McGregor and Nelson, 2019). The striatum integrates excitatory (glutamatergic) cortical inputs and directs them towards the BG output nuclei (i.e., globus pallidus internal (GPi)/substantia nigra pars reticulata (SNr)), and ultimately, to the thalamus to regulate motor coordination (DeLong, 1990; Graybiel et al., 1994). This relay happens either from the direct (Go) pathway or the indirect (NoGo) pathway—mediated by the globus pallidus external (GPe)-STN network—which opposingly modulate the BG output nuclei. 2) The globus pallidus is composed of external (GPe) and internal (GPi) segments. 3) The substantia nigra includes the pars compacta (SNc; comprising dopaminergic (DAergic) cells) and pars reticulata (SNr) segments. In addition to SNc, the ventral tegmental area (VTA) is another source of DAergic neurons in the midbrain. DAergic inputs from the VTA modulate the activity of neurons within the NAc that is associated with reward-related processes (Schultz, 2002), whereas the DA signaling from the SNc is linked to motor control (McGregor and Nelson, 2019). 4) The STN receives projections from GPe (via the indirect or NoGo pathway) and cortex (via the hyperdirect or global NoGo pathway), and projects to GPe as well as GPi/SNr.

The striatal neurons can be mostly characterized as the inhibitory (gamma-aminobutyric acid; GABAergic) medium spiny neurons (MSNs) (Gerfen et al., 1990). However, the presence of cholinergic and other GABAergic interneurons in the striatum can modify the intrinsic properties of MSNs (Shen et al., 2007), and in this way, affect the cortico-striatal transmission (Wang et al., 2006; Fino et al., 2008). Both direct and indirect pathways are under the influence of dopamine (DA) released from the SNc DAergic neurons (Calabresi et al., 2007; Surmeier et al., 2007; Pawlak and Kerr, 2008; Shen et al., 2008). Subpopulations of MSNs can be roughly categorized by their DAergic receptor expression properties (Gerfen et al., 1990): D1-type MSNs are attributed to the direct pathway and D2-type MSNs act in the indirect pathway. In the classical model of the BG, DA opposingly modulates the direct and indirect pathways, i.e., activity of neurons in the direct pathway is facilitated by the activation of D1 receptors (D1Rs), whereas neurons in the indirect pathway are inhibited by the activation of D2 receptors (D2Rs) (Surmeier et al., 2007). This leads to an imbalance of firing rates of the striatal neurons acting in these two competing motor pathways. Accordingly, one can hypothesize that DA loss may suppress neuronal activity in the direct pathway but promote activity in the indirect pathway (Gerfen and Surmeier, 2011). In addition, incoming cortico-STN input via the hyperdirect pathway (see Figure 1) is altered in PD (Chu et al., 2017) which can disturb the excitation-inhibition balance towards the STN. The interaction between the hyperdirect and the indirect pathway can contribute to the emergence of pathological activity-connectivity states in the GPe-STN network (Chu et al., 2015).

The classical rate-based view of the functional structure of direct and indirect pathways, however, was later challenged by contradictory observations (Calabresi et al., 2014; Spix and Gittis, 2020). For example, in a study with careful spike counting, Valsky et al. (2020) revealed contradictory findings regarding neuronal firing rates and activity patterns in the striatum of PD patients. The rate model predicts that opposing firing rate changes of striatal neurons in the direct and indirect pathways following DA loss ultimately leads to overly synchronized activity in downstream populations accompanied with pronounced oscillations (Spix and Gittis, 2020). However, Valsky et al. (2020) found no particular difference between the firing rates (or patterns of activity) obtained from striatal neurons of PD patients and those found in healthy non-human primates. In fact, they were unable to discriminate two populations that could be representative of hypoactive D1-MSNs and hyperactive D2-MSNs (Valsky et al., 2020).

While the classical model provides insights into fundamental BG function, other models of BG may shed light on different aspects of BG function. In the parallel circuit model for instance, distinct limbic, associative and sensorimotor channels are supposedly responsible for information transmission through downstream BG subnetworks (McGregor and Nelson, 2019). DA loss can impair motor regions of the striatum, but how nonmotor channels are affected by DA deficiency is still elusive. In this regard, some studies linked the DA dysfunction to psychiatric disorders such as schizophrenia and addiction (Gray et al., 1995; Di Chiara et al., 1999; Maia and Frank, 2011; Maia and Frank, 2017; Solinas et al., 2019). On the contrary, the center-surround model emphasizes the opposing DA modulation of striatal MSNs in the direct and indirect pathways (like in the classical model), but brings forward the complementary roles that these pathways might play in movement initiation supported by the concurrent activation of both pathways during action initiation (Cui et al., 2013). In particular, according to the center-surround model the selection of one action and the related neuronal activity requires simultaneous suppression of other, similar or competing actions and related activity patterns (McGregor and Nelson, 2019).

In this review, we focus on DA-related plasticity processes, in particular, DA-related STDP involved in PD pathophysiology. DA loss in experimental animal models of PD initiates a series of malfunctions leading to the emergence of pathological changes in certain subnetworks of the BG (Mallet et al., 2006; Fino et al., 2007; Mallet et al., 2008b; Lemos et al., 2016). Such pathological changes ultimately result in the dysfunction of the cortico-basal ganglia-thalamo-cortical (CBGTC) loop that is implicated in movement disorders (DeLong and Wichmann, 2007). For instance, the appearance of abnormal, excessive neuronal synchronization in the beta band (15–30 Hz) in the STN is a hallmark of PD (Nini et al., 1995; Brown et al., 2001; Levy et al., 2002; Hammond et al., 2007; Mallet et al., 2008a). The notion that DA loss may be one of the primary origins of movement abnormalities associated with PD comes from observations that DA replacement therapies can control motor symptoms in models of PD by reducing beta oscillations (Kühn et al., 2006).

Experiments performed in rat/mouse slices verified that PD symptoms gradually appear after DA depletion within a couple of weeks (Mallet et al., 2008b; Fan et al., 2012; Chu et al., 2015), suggesting that plasticity mechanisms may potentially be involved in the long-term emergence of pathological states. In fact, the synaptic strengths in different pathways of the BG are mediated by the STDP rule where its temporal window for LTP/LTD induction is critically gated by DA (Calabresi et al., 1997; Centonze et al., 1999; Picconi et al., 2003; Pawlak and Kerr, 2008). Therefore, DA modulation of STDP (and possibly other plasticity mechanisms) can affect BG connectivity and consequently its dynamics (Frémaux and Gerstner, 2016). Depletion of DA might lead to a dysfunction of the activity-dependent modification of synaptic connectivity and can contribute to the emergence of pathological states, with strongly synchronized activity and strongly connected neurons, which is a hallmark of a number of neurological disorders such as PD (Nini et al., 1995; Hammond et al., 2007). The notion of DA depletion-induced changes of (synaptic) plasticity deserves to be focused on.

In fact, the role of STDP and other plasticity mechanisms in the dynamical and structural regulation of the CBGTC loop under normal as well as parkinsonian conditions can be considered from multiple viewpoints: 1) DA massively modulates the temporal contrast of STDP such that in DA-depleted pathological conditions synaptic plasticity fails to function normally and leads to undesired modifications of the synapses due to the deformation of the STDP learning window or blockade of LTP/LTD (Kerr and Wickens, 2001; Pawlak and Kerr, 2008). In addition, concurrent action of the classical STDP, structural plasticity and homeostatic plasticity may change the net performance of the CBGTC loop in a complex manner. 2) Cascades of maladaptive or compensatory changes following DA loss may lead to BG circuit dysfunction (Blesa et al., 2017; Villalba and Smith, 2010). This review aims at highlighting the key role of plasticity mechanisms, especially STDP, in the emergence and stabilization of different regimens of BG circuit dysfunction. 3) Computationally, the emergence of multistable network dynamics is one of the potential consequences of STDP (Madadi Asl et al., 2018a). By simultaneous modulation of activity-connectivity patterns, STDP can mediate a repertoire of qualitatively different network regimes, ranging from pathological states with strong synchrony and strong synaptic connectivity to normal states with weaker synchrony and weaker synaptic connectivity (Madadi Asl et al., 2017; Madadi Asl et al., 2018a).

In what follows we discuss different aspects of STDP and other plasticity mechanisms in the light of experimental findings and computational modeling studies. First, we review experimental studies on DA-mediated STDP and the potential role of other plasticity mechanisms. Then we focus on the dynamical consequences of STDP as regards the stabilization of healthy or disease states. To this end, we review a number of experimental observations on how DA depletion can trigger plasticity-induced maladaptive or compensatory processes at structural and dynamical levels, leading to dysfunction of different BG pathways. Finally, the implications of these altered patterns of neuronal activity and synaptic connectivity are discussed in relation to PD.

## 2 Dopaminergic Modulation of Spike-Timing-Dependent Plasticity

Following lesions of midbrain DAergic neurons in animal models of PD, it takes a couple of weeks for parkinsonian abnormal activity to emerge and stabilize in the GPe-STN circuit (Mallet et al., 2008b; Fan et al., 2012; Chu et al., 2015), suggesting that plasticity mechanisms may be involved in the emergence of long-lasting pathological changes in the BG after DA loss (Day et al., 2006; Taverna et al., 2008; Fan et al., 2012; Chu et al., 2017) (for a review see (Chu, 2020)). This was further validated by experimental observations suggesting that motor-related functions of the direct and indirect pathways crucially depend on the form of synaptic plasticity expressed by selective activation or blockade of DA receptors (Calabresi et al., 1996; Calabresi et al., 1997; Centonze et al., 1999).

At the cellular level, DA modulates synaptic integration and synaptic transmission by regulating the intrinsic properties of cells, i.e., the excitability of the pre- and postsynaptic neurons, probability of neurotransmitter release and receptors’ response to neurotransmitters by selective activation of D1- or D2-like receptors (Tritsch and Sabatini, 2012; Chu, 2020). These changes can be regarded as metaplasticity mechanisms that ultimately determine functional implications of synaptic plasticity at higher levels by modification of the synaptic strengths via adjusting the evoked excitatory or inhibitory postsynaptic potentials (EPSPs or IPSPs) (Calabresi et al., 1997; Centonze et al., 1999; Picconi et al., 2003; Shen et al., 2008). In this way, DA controls the induction of long-term synaptic changes by adjusting LTP/LTD in a preferred direction (Calabresi et al., 1997; Centonze et al., 1999; Picconi et al., 2003).

**FIGURE 2:**
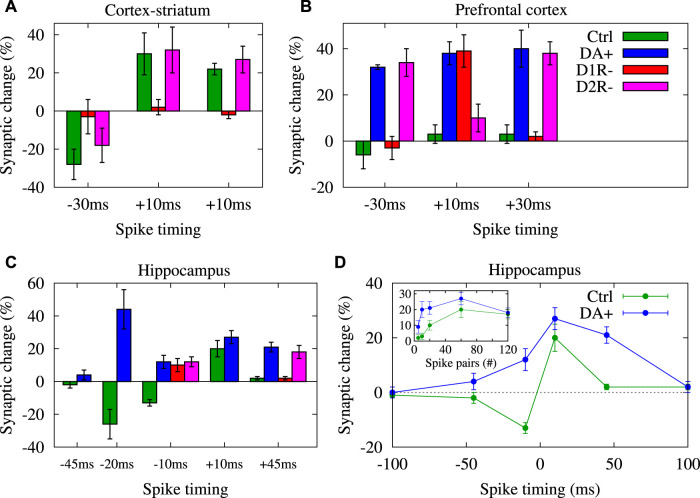
DA shapes STDP temporal window for LTP/LTD induction. Visualization of data presented in Table 1 for synapses in cortex-striatum **(A)**, hippocampus **(B)** and PFC **(C)** of rats/mice slices, respectively in control (ctrl), DA application (DA+), D1R blocked (D1R-) and D2R blocked (D2R-) conditions. **(D)** Conversion of timing-dependent LTD (t-LTD) into timing-dependent LTP (t-LTP) for post-pre (negative) spike timings and enhancement of t-LTP for pre-post (positive) pairings in the presence of DA vs. the corresponding pairing experiments in the control condition in rat hippocampal slices for 60 spike pairs delivered at 1 Hz. (Inset to **D**) Reduction of threshold required for the t-LTP induction in the presence of DA in comparison to the corresponding control experiments in same slices for different number of repetitive spike pairs (at 1 Hz) with Δ*t* = + 10 ms. Panel **(D)** is adapted from Zhang et al. (2009) with no permission required.

Stimulus pairing experiments at the cortico-striatal synapses onto MSNs of rat slices in control (intact) conditions showed a bidirectional and temporally asymmetric STDP window (i.e., pre-post pairing (Δ*t* > 0) induced LTP, whereas post-pre pairing (Δ*t* < 0) induced LTD) (Pawlak and Kerr, 2008). The sensitivity of the STDP window to the pairing timing of striatal action potentials relative to cortical inputs was such that the spike timing interval for LTP induction was relatively small and close to zero, while the spike timing interval for LTD induction was broad and only observed for relatively long time differences, e.g., Δ*t* = −30 ms (EPSP amplitude indicated in rows no. 1, 2 in Table 1; also see Figure 2A) (Pawlak and Kerr, 2008). Interestingly, this profile of cortico-striatal STDP resembles classical STDP profiles that were measured experimentally in cortical (Markram et al., 1997; Froemke and Dan, 2002) and hippocampal (Bi and Poo, 1998) slices characterized by a significant LTP in small spike timings and a long-tailed temporal window for LTD where the change of the synaptic strengths exponentially decays with the spike timing.

**TABLE 1:** DAergic modulation of STDP observed by stimulus pairing experiments in brain slices of rats/mice.

	No	EPSP ampl. (%)	Δ*t* (ms)	*n* (#)	Condition	center
Cortex-striatum	1	+30 ± 11	+10	11	Control	Pawlak and Kerr (2008)
	2	−28 ± 08	−30	12		
	3	+02 ± 04	+10	09	D1R/D5R blocked	
	4	−03 ± 09	−30	07		
	5	+32 ± 12	+10	08	D2R blocked	
	6	−18 ± 09	−30	05		
	7	+22 ± 03	+10	06	Control	Kerr and Wickens (2001)
	8	−02 ± 02	+10	05	D1R/D5R blocked	
	9	+27 ± 07	+10	04	D2R blocked	
Prefrontal cortex	10	+03 ± 04	+10	10	Control	Xu and Yao (2010)
	11	+03 ± 04	+30	07		
	12	+38 ± 05	+10	10	DA application	
	13	+40 ± 08	+30	08		
	14	+39 ± 07	+10	08	D1R blocked	
	15	+02 ± 02	+30	08		
	16	+10 ± 06	+10	08	D2R blocked	
	17	+38 ± 05	+30	08		
	18	−06 ± 06	−30	10	Control	Ruan et al. (2014)
	19	+32 ± 01	−30	08	DA application	
	20	−03 ± 05	−30	09	D1R blocked	
	21	+34 ± 06	−30	08	D2R blocked	
Hippocampus	22	+20 ± 05	+10	13	Control	Zhang et al. (2009)
	23	+02 ± 01	+45	12		
	24	−13 ± 02	−10	12		
	25	−02 ± 02	−45	05		
	26	+27 ± 04	+10	06	DA application	
	27	+21 ± 03	+45	07		
	28	+12 ± 04	−10	07		
	29	+04 ± 03	−45	05		
	30	+02 ± 01	+45	06	D1R/D5R blocked	
	31	+10 ± 04	−10	05		
	32	+18 ± 04	+45	06	D2R blocked	
	33	+12 ± 03	−10	05		
	34	−26 ± 09	−20	09	Control	Brzosko et al. (2015)
	35	+44 ± 12	−20	06	DA application	

However, these long-term synaptic changes at the cortico-striatal connections critically depend on the activation of DA receptors (Calabresi et al., 2007; Pawlak and Kerr, 2008; Rueda-Orozco et al., 2009) such that blocking DA D1R/D5R prevents both LTP (cf. EPSP amplitude in rows no. 3 and no. 1 in Table 1; D1R/D5R blocked: + 2 ± 4% vs. control: + 30 ± 11% when Δ*t* = + 10 ms; also see Figure 2A) and LTD (cf. EPSP amplitude in rows no. 4 and no. 2 in Table 1; D1R/D5R blocked: −3 ± 9% vs. control: −28 ± 8% when Δ*t* = −30 ms; also see Figure 2A) (Pawlak and Kerr, 2008). The blockade of DA D2R, however, does not significantly change the long-term outcome of cortico-striatal STDP where the change of the EPSP amplitude relative to the baseline (measured 20–30 min after the STDP protocol) remained relatively the same for the D2R blocked vs. control condition (cf. rows no. 5, 6 and no. 1, 2 in Table 1; also see Figure 2A) (Pawlak and Kerr, 2008). These results were in accordance with previous findings that DA D1R/D5R, but not D2R, activation is required for LTD induction by HFS (100 Hz) at the cortico-striatal connections of rat slices (see rows no. 7–9 in Table 1; Figure 2A) (Kerr and Wickens, 2001).

DA modulation of the temporal window required for LTP/LTD induction at cortico-striatal connections of rats or mice was reported by similar experiments. Some of these findings were contradictory. For instance, in the control condition, HFS of cortico-striatal fibers within the striatum induced LTD of the synaptic strengths (Calabresi et al., 1992a; Calabresi et al., 1992b; Calabresi et al., 1997) that was abolished when DA D1R/D2R was blocked (Calabresi et al., 1992a; Calabresi et al., 1992b; Calabresi et al., 1997). Furthermore, unilateral lesion of the nigro-striatal DAergic pathway in rat slices by 6-hydroxydopamine (6-OHDA) blocked HFS-induced LTP (stimulation applied to the cortex) (Centonze et al., 1999) and LTD (stimulation applied to the striatum) (Calabresi et al., 1992b; Calabresi et al., 1992a). Coadministration of DA D1R and D2R agonists, but not a single class of DA receptor agonists alone, can restore LTD implying that co-activation of D1R and D2R is necessary for cortico-striatal LTD (Calabresi et al., 1992b; Calabresi et al., 1992a). Surprisingly, recordings from D2R-null mouse showed that HFS of wild-type mouse cortico-striatal synapses induced LTP instead of LTD (seen in control D2R-intact condition) that can be reversed into LTD by D2R agonists showing that D2Rs may determine the direction of long-term synaptic changes in the cortico-striatal pathway (Calabresi et al., 1997).

In general, these results suggest that activation of DA D1Rs promotes LTP, whereas the activation of DA D2Rs promotes LTD. This may imply that STDP is unidirectional at a given cortico-striatal synapse since DA D1Rs and D2Rs are expressed in different MSNs, but experimental findings suggested that both LTP and LTD can be induced selectively by HFS (at 100 Hz) or low-frequency stimulation (LFS; 1 Hz) in rat cortico-striatal synapses ensuring bidirectional Hebbian forms of STDP (Fino et al., 2005; Yamawaki et al., 2012). HFS of cortical afferents can restore LTP of cortico-striatal synapses in L-3,4-dihydroxyphenylalanine (L-DOPA)-treated parkinsonian rats (that showed impaired cortico-striatal LTP) (Picconi et al., 2003). Following HFS, the delivery of LFS (1–2 Hz) can turn the synaptic strengths to pre-LTP levels (a process termed as depotentiation) (Picconi et al., 2003). Interestingly, LFS fully reversed LTP in control (intact) and non-dyskinetic rats, but dyskinetic rats failed to produce depotentiation (Picconi et al., 2003). Furthermore, cortico-striatal afferent stimulation (at 5 Hz) revealed that both D1- and D2-expressing MSNs can display LTP (at pre-post or positive pairing) and LTD (at post-pre or negative pairing) that guarantees a bidirectional Hebbian form of STDP (Shen et al., 2008) which is necessary for shaping the synaptic strengths, e.g., during associative learning (Schultz et al., 1997). It is also shown that DA works in harmony with glutamate and adenosine to induce long-term changes of cortico-striatal synapses that poses further complexity on the role of D1Rs and D2Rs in the induction of STDP (Shen et al., 2008).

DAergic modulation of STDP was also verified by stimulus pairing experiments in slices of other brain regions in rats/mice. In prefrontal cortex (PFC) synapses of mice, DA application enabled t-LTP in intact slices by stimulation (at 0.1 Hz) for Δ*t* = + 10 ms (cf. rows no. 10 and 12 in Table 1; control: + 3 ± 4*%*, *n* = 10 vs. DA application: + 38 ± 5*%*, *n* = 10; also see Figure 2B) by the activation of D2Rs (Xu and Yao, 2010). In particular, blockade of D2Rs abolished t-LTP (+10 ± 6*%*, *n* = 8), whereas blockade of D1Rs made no difference (+39 ± 7*%*, *n* = 8) suggesting that D2Rs, but not D1Rs, gate t-LTP at a narrow spike timing (i.e. Δ*t* = + 10 ms) (Xu and Yao, 2010). However, at an extended timing interval (i.e. Δ*t* = + 30 ms), DA application in slices with blocked GABAergic transmission induced robust t-LTP (cf. rows no. 11 and 13 in Table 1; control: + 3 ± 4%, *n* = 7 vs. DA application: + 40 ± 8%, *n* = 8; also see Figure 2B) that was blocked by D1R antagonist (+2 ± 2%, *n* = 8), but not by D2R antagonist (+38 ± 5%, *n* = 8) (Xu and Yao, 2010). In addition, application of D1R agonist enabled t-LTP induction (+32 ± 6%, *n* = 8) implying that activation of D1Rs can mimic DA to enable t-LTP at Δ*t* = + 30 ms which enhances the associability of coincident stimuli for positive (pre-post) pairings (Xu and Yao, 2010). However, in intact slices no t-LTP was found (−2 ± 4%, *n* = 7) at Δ*t* = + 30 ms unless DA was applied (+36 ± 7*%*, *n* = 8). t-LTP was completely abolished by either D1R antagonist (0 ± 1%, *n* = 6) or D2R antagonist (0 ± 5%, *n* = 5), indicating that D1Rs and D2Rs jointly enable t-LTP at the extended pairing timing (i.e., Δ*t* = + 30 ms) in physiological conditions (Xu and Yao, 2010).

Consistent with those observations obtained from cortico-striatal connections of mouse (Calabresi et al., 1997) and rat hippocampal synapses (Zhang et al., 2009), conversion of t-LTD into t-LTP for post-pre (negative) pairings was reported in mouse PFC synapses in intact inhibitory transmission condition (Ruan et al., 2014). In this case, stimulation (at 0.1 Hz) failed to induce t-LTD at Δ*t* = −30 ms in the control condition (−6 ± 6%, *n* = 10), but induced significant t-LTP (+32 ± 1%, *n* = 8) when DA was applied (Ruan et al., 2014). This t-LTP was blocked by D1R antagonist (−3 ± 5%, *n* = 9), but not by D2R antagonist (+34 ± 6%, *n* = 8), suggesting that unlike pre-post (positive) pairings D2Rs do not contribute to this DA-enabled t-LTP induced at post-pre (negative) pairings (Ruan et al., 2014).

Qualitatively similar results were experimentally observed in hippocampal synapses. In particular, DA converts the timing-dependent (at 1 Hz) synaptic depression (t-LTD) into potentiation (t-LTP) in rat hippocampal synapses via the activation of DA D1Rs, most significantly at Δ*t* = −10 ms shown in Figure 2D (cf. rows no. 24 and 28 in Table 1; control: −13 ± 2%, *n* = 12 vs. DA application: +12 ± 4%, *n* = 7; also see Figure 2C) (Zhang et al., 2009). Comparable observations were made in mouse hippocampal synapses at Δ*t* = −20 ms (cf. rows no. 34 and 35 in Table 1; control: −26 ± 9%, *n* = 9 vs. DA application: + 44 ± 12%, *n* = 6; also see Figure 2C) for 100 spike pairs at 0.2 Hz (Brzosko et al., 2015). These observations are consistent with similar findings obtained from rat cortico-striatal synapses where DA application reversed LTD (control: − 27 ± 4%, *n* = 7) into LTP (DA application: + 21 ± 7%, *n* = 7) in response to cortical HFS (6 trains of 20 pulses at 100 Hz) (Wickens et al., 1996). Furthermore, DA application reduces the pairing repetition threshold required for t-LTP induction in rat hippocampal synapses (inset to Figure 2D, blue) compared to the control condition (inset to Figure 2D, green) when different numbers of spike pairs (at 1 Hz) were delivered to the pre- and postsynaptic neurons with Δ*t* = + 10 ms (Zhang et al., 2009).

Although the experimental findings may be contradictory in some cases, they carry a clear and concise message: The classical temporally asymmetric form of the STDP learning window can be crucially reshaped by the presence or absence of DA. The classical STDP profile is characterized by the significant potentiation of synapses for narrow pre-post (positive) pairings and a long-tailed regime for the depression of synapses at post-pre (negative) pairings (Bi and Poo, 1998), ensuring a competitive Hebbian learning mechanism (Song et al., 2000). In this way, neurons with correlated activity develop stronger synapses, whereas synapses representing less effective (uncorrelated) inputs are weakened through a competitive modification of the synaptic strengths (Song et al., 2000). However, experimentally observed deformation of the STDP temporal window by DA in comparison to the classical STDP profile (e.g., blockade of LTP/LTD or conversion of LTD into LTP for post-pre pairing) disturbs the temporal asymmetry of the STDP learning window and the competition between synapses. This may contribute to the changes of the structure and dynamics in pathological states, e.g., following DA loss in the parkinsonian state (Dupuis et al., 2013; Chu, 2020).

## 3 Dopaminergic Modulation of Other Plasticity Mechanisms

### 3.1 Structural Plasticity

Various plasticity mechanisms govern the evolution of connectivity and activity patterns at multiple levels (Zenke et al., 2015). At the functional level, strength and shape of existing synaptic connections is regulated by, e.g., Hebbian forms of the STDP rule according to the spike timing of neurons (Markram et al., 1997; Bi and Poo, 1998). At the structural level, however, generation and deletion of synaptic connections is controlled by structural plasticity mechanisms (Butz et al., 2009). In addition to the modulation of extent or shape of dendrites and axons, structural plasticity also induces structural modifications in spines and boutons supported by *in vivo* experiments in mouse neocortex neurons (De Paola et al., 2006) and *in vitro* experiments in rat hippocampal cultures (Oh et al., 2015). Structural reshaping of connections between neurons is implicated in the stabilization of synapses for memory formation (Xu et al., 2009) and may determine adaptive and maladaptive responses of the nervous system to central and peripheral lesions (Yamahachi et al., 2009).

Morphological changes of the dendrites of MSNs induced by structural plasticity can be linked to neurodegeneration processes. For instance, denervation of DAergic neurons modulates striatal structural plasticity and can lead to the loss of dendritic spines in striatal MSNs in the direct and indirect pathways (Villalba et al., 2009; Suárez et al., 2014; Suárez et al., 2016; Suarez et al., 2020) (for a review see (Fiala et al., 2002; Villalba and Smith, 2010)). Postmortem analysis of PD patients revealed a reduction of the dendrite length and the number of dendritic branches, a notable loss of dendritic spines and an approximately 27% decrease in dendritic spine density of striatal MSNs (Stephens et al., 2005; Zaja-Milatovic et al., 2005).

Qualitatively similar observations were made in animal models of PD. For instance, in the 6-OHDA rat model of PD spine density of striatal MSNs was decreased by approximately 20% compared to controls (Ingham et al., 1989; Ingham et al., 1993). Both D1R-MSNs and D2R-MSNs in 6-OHDA-lesioned wild-type mice showed a decreased spine density in severely denervated striatal regions (Suárez et al., 2014). Chronic L-DOPA treatment restored spine density in D2R-MSNs but not in D1R-MSNs (Suárez et al., 2014). Similarly, significant spine loss (30%–50*%*) was observed in striatal MSNs of 1-methyl-4-phenyl-1,2,3,6-tetrahydropyridine (MPTP)-treated parkinsonian non-human primates (Villalba et al., 2009). The modulation of morphological properties of MSNs in the direct and indirect pathways is accompanied by changes of the strength of synaptic transmission (Suárez et al., 2016), and consequently, firing rates of affected MSNs (Suárez et al., 2014).

It was argued that the genetic inactivation of D1Rs in mice can reduce the length of the dendritic branches and the spine density in MSNs in both direct and indirect pathway (Suarez et al., 2020). Although the effect was more severe in MSNs acting in the direct pathway, this observation reveals that D1Rs not only play a key role in the preservation of spine plasticity in the direct pathway but also modulate MSNs in the indirect pathway (Suarez et al., 2020). However, whether spine pruning occurs in MSNs of both direct and indirect pathway is still under debate. While the results of some experiments show that spine pruning merely occurs in indirect pathway MSNs (Day et al., 2006), other experiments suggest that MSNs of both direct and indirect pathway undergo spine pruning (Villalba et al., 2009; Suárez et al., 2014; Suárez et al., 2016). One possible explanation for this discrepancy may be the time interval between the lesion occurrence and the assessment of spine loss (Graves and Surmeier, 2019).

Structural plasticity does not necessarily follow Hebbian or anti-Hebbian paradigms of plasticity (Butz et al., 2009). However, it has been theoretically argued that some forms of structural plasticity can be phenomenologically related to Hebbian plasticity according to their activity dependency, i.e., the connectivity is strengthened, with number of synapses being increased, during highly correlated neuronal activity and vice versa (Helias et al., 2008; Fauth and Tetzlaff, 2016). In fact, experimental studies on hippocampal pyramidal neurons have suggested that alterations in spine volume is strongly correlated with the amplitude of excitatory postsynaptic currents (EPSCs) (Matsuzaki et al., 2001), likely mediated by DA action (Yagishita et al., 2014). This suggests that functional synaptic plasticity (e.g. STDP) and structural plasticity may be tightly related and cooperate to determine the dynamics and structure of brain networks. Indeed, by employing a combined model of structural plasticity and STDP computational studies were able to explain experimentally observed multiple synaptic contacts (Deger et al., 2012; Deger et al., 2018) or non-random features of connectivity pattern in cortex (Zheng et al., 2013).

### 3.2 Homeostatic Plasticity

Hebbian synaptic plasticity is intrinsically a positive feedback process with a propensity for runaway neuronal activity by excessive growth of the synaptic strengths, and hence, requires stabilization by other compensatory mechanisms. The stabilization of these changes at a functional set point underlies activity-dependent homeostatic regulation of the synaptic strengths in the network based on a negative feedback process (Turrigiano and Nelson, 2004; Turrigiano, 2008; Pozo and Goda, 2010). This requires dynamic adaptation of the network activity pattern, e.g., suggested by the Bienenstock-Cooper-Munro (BCM) plasticity rule via introducing a sliding threshold for neuronal activity (Bienenstock et al., 1982) that can be related to the STDP rule (Izhikevich and Desai, 2003).

The exact mechanism underlying mutual interaction between Hebbian plasticity and homeostatic plasticity, however, has remained elusive due to experimental challenges. It has been suggested that homeostatic plasticity may function on a slower time-scale than that of Hebbian plasticity so that they may not interfere with each other (Kirov and Harris, 1999; Turrigiano, 2012). This notion was challenged by computational modeling studies (Zenke et al., 2013; Zenke et al., 2017) further supported by experimental findings (Keck et al., 2011; Li et al., 2014), implying that such slow operation of homeostatic plasticity may not be adequate to resolve instabilities induced by the Hebbian plasticity. Alternatively, this issue can be resolved by assuming that Hebbian plasticity must attain its own stable steady state that is not disturbed by the slow dynamics of homeostatic plasticity (Toyoizumi et al., 2014). This was theoretically implemented in a biophysically plausible model where the synaptic strength was the product of two factors separately controlled by homeostatic plasticity and Hebbian plasticity (Toyoizumi et al., 2014). This model successfully reproduced experimental findings obtained in visual cortex (Toyoizumi et al., 2014).

In an attempt to address the interaction between Hebbian and homeostatic plasticity, a number of experimental studies explored the functional and structural implications of homeostatic plasticity at dendritic spines of mouse hippocampal neurons (Kirov and Harris, 1999; Hobbiss et al., 2018). These findings revealed that chronic activity blockade can lead to spine growth where the synaptic strengths are then regulated by Hebbian plasticity according to volume changes of spines (Hobbiss et al., 2018). Apart from interactions between Hebbian synaptic plasticity and homeostatic plasticity, loss or remodeling of dendritic spines in striatal MSNs in PD condition observed in several experiments has been interpreted as homeostatic regulation of structural plasticity (Day et al., 2006; Butz et al., 2009; Thibault et al., 2016; Villalba and Smith, 2018). Although several factors contribute to the neurodegeneration process in PD, the involvement of homeostatic mechanisms in the regulation of structural plasticity in striatal MSNs can be traced back to the degeneration of nigro-striatal DAergic neurons (Azdad et al., 2009; Suárez et al., 2014; Suárez et al., 2016).

In fact, DA depletion in animal models of PD leads to compensatory or maladaptive transformation of the BG circuitry. Alternation of the morphology of striatal MSNs is one of the early changes following DA loss. Particularly, severe loss of dendritic spines (Azdad et al., 2009; Thibault et al., 2016; Villalba and Smith, 2018) and the reshaping of axo-spinous glutamatergic synapses (Day et al., 2006) are closely correlated with the degree of DA neurodegeneration, but not the extent of PD motor symptoms. DA depletion results in an increase in MSNs’ intrinsic excitability despite loss of dendritic spines accompanied with the significant suppression of their excitatory synaptic inputs (Fino et al., 2007; Azdad et al., 2009). These observations suggest that spine pruning of striatal MSNs and then enhancement of their intrinsic excitability may be interpreted as a form of homeostatic plasticity that counteracts DA deficiency and preserves the responsiveness of surviving synapses (Azdad et al., 2009; Thibault et al., 2016; Villalba and Smith, 2018). On the other hand, reshaping of axo-spinous cortico-striatal synapses or selective elimination of glutamatergic synapses on striato-pallidal neurons may be assumed as a maladaptive plasticity that pathologically changes the synaptic strengths resulting in the changes of firing rates or firing patterns of MSNs in the striatum or its descending pathways in parkinsonian state (Day et al., 2006; Surmeier et al., 2007).

Although various forms of Hebbian plasticity, structural plasticity or homeostatic plasticity have been separately investigated previously, their interactions have not yet been appropriately unveiled (at least experimentally). This might be due to practical limitations in experimental designs. However, complex interactions between these (synaptic) plasticity paradigms at different levels may be key to the emergence of compensatory and maladaptive changes that occur after DA depletion in the parkinsonian BG. The functional and structural consequences of these changes deserve to be addressed.

## 4 The Role of Plasticity in Parkinson’s Disease

PD is a progressive brain disorder that is related to multi-systemic neurodegeneration (Surmeier et al., 2017; McGregor and Nelson, 2019). A number of PD symptoms are associated with widespread neuronal loss, whereas other symptoms are linked to abnormal neuronal activity (McGregor and Nelson, 2019). Motor symptoms of PD are linked to DA loss followed by BG circuit dysfunction (Brown et al., 2001; Mallet et al., 2008b). Structural and dynamical changes following DA loss eventually lead to abnormal neuronal activity including the hyperactivity of MSNs in the indirect pathway, and consequently the STN (Levy et al., 2002; Mallet et al., 2008a; Mallet et al., 2008b). This excessive synchronized activity subsequently propagates to the BG output nuclei (i.e., GPi/SNr) that contributes to impaired motor coordination observed in PD (Levy et al., 2002; Meissner et al., 2005; Kühn et al., 2006).

Among the great number of computational and experimental studies on PD, the effect of plasticity mechanisms on the emergence of PD-related pathological changes is not adequately appreciated. However, (synaptic) plasticity may be considered as the missing link between structural/functional changes following DA loss (i.e., groups a-c in Table 2, as described below) and its dynamical consequences (i.e., group d in Table 2) in the PD condition. In fact, DA deficiency dramatically alters the temporal profile of STDP at cortico-striatal synapses leading to the change of synaptic strengths in an undesired direction due to blockade of LTP/LTD (Wickens et al., 1996; Centonze et al., 1999; Kerr and Wickens, 2001; Pawlak and Kerr, 2008). Consequently, both the number of synapses and their strength in the indirect pathway are increased (Fan et al., 2012; Lemos et al., 2016), whereas the number of synapses in the hyperdirect pathway is decreased (but not necessarily the strength of the corresponding excitatory input) (Chu et al., 2017). These plasticity-induced modifications may ultimately change the firing pattern of the GPe and STN cells and promote abnormal rhythmogenesis in the GPe-STN circuit in the PD condition (Sharott et al., 2005; Mallet et al., 2008a; Mallet et al., 2008b; Kita and Kita, 2011). This can crucially affect action selection and specific movement tasks by reshaping the normal competition between the direct and indirect pathways at the level of the BG output nuclei (Leblois et al., 2006; Calabresi et al., 2014).

**TABLE 2:** Different changes following DA depletion in different pathways of the BG that were experimentally observed in rats/mice slices.

No	Observation	Pathway/region	References
Group (a)	Enhanced excitability of MSNs	Cortico-striatal	Calabresi et al. (1993)
			Fino et al. (2007)
			Azdad et al. (2009)
		Striato-pallidal	Shen et al. (2007)
	Enhanced excitability of cholinergic cells	Cortico-striatal	Fino et al. (2007)
	Reduced excitability of GABAergic cells		
Group (b)	Blockade of pre-post LTP	Cortico-striatal	Centonze et al. (1999)
			Kerr and Wickens (2001)
			Pawlak and Kerr (2008)
	Blockade of post-pre LTD	Cortico-striatal	Calabresi et al. (1992b)
			Calabresi et al. (1997)
			Pawlak and Kerr (2008)
	Conversion of post-pre LTP into LTD	Cortico-striatal	Wickens et al. (1996)
	Loss of bidirectional STDP	Cortico-striatal	Picconi et al. (2003)
			Shen et al. (2008)
Group (c)	Depression of synapses between MSNs	Cortico-striatal	Taverna et al. (2008)
	Loss of glutamatergic synapses on MSNs	Striato-pallidal	Day et al. (2006)
	Enhanced GABAergic transmission	Striato-pallidal	Lemos et al. (2016)
	Enhanced synaptic transmission	Pallido-pallidal	Miguelez et al. (2012)
	Proliferation of synapses	Pallido-subthalamic	Fan et al. (2012)
			Chu et al. (2015)
	Loss of synaptic connections	Cortico-subthalamic	Chu et al. (2017)
Group (d)	Suppressed/enhanced firing rate	Striatum	Kita and Kita (2011)
	of D1R/D2R MSNs		Ryan et al. (2018)
	Enhanced coherency/bursting/pausing	GPe	Mallet et al. (2008a)
	of firing pattern; suppressed firing rate		Kita and Kita (2011)
	Exaggerated oscillations at 15–30 Hz	STN	Mallet et al. (2008b)
			Sharott et al. (2005)

STDP-induced long-term synaptic changes were experimentally observed in the BG, e.g., between striatal MSNs by repetitive use of HFS on the cortical excitatory afferents projecting onto the MSNs in mouse and rat slices (Calabresi et al., 1992b; Calabresi et al., 1996; Mahon et al., 2004; Fino et al., 2005; Kreitzer and Malenka, 2008; Di Filippo et al., 2009) or within slices of rat STN neurons (Shen et al., 2003). In fact, MSNs discharge in response to strongly correlated cortical inputs. Striatal MSNs thus are able to discriminate relevant information from noisy cortical input by coincidence detection of activity (Fino et al., 2010). This is an intrinsic property of STDP that has been observed earlier by stimulus pairing experiments in cortical (Markram et al., 1997; Sjöström et al., 2001; Froemke and Dan, 2002) and hippocampal (Magee and Johnston, 1997; Bi and Poo, 1998) slices of mice and rats: Modification of the synaptic strengths critically depends on the coincidence of pre- and postsynaptic spike pairs within several milliseconds.

Experimental PD models of mice and rats have shown that the synaptic transmission (accompanied by changes in the number of connections or strength of synapses) in the striato-pallidal (Lemos et al., 2016), pallido-pallidal (Miguelez et al., 2012) and pallido-subthalamic (Fan et al., 2012) pathways profoundly increased following DA depletion. This may further amplify pathologically correlated neuronal activity in the involved nuclei. Assuming that maladaptive (synaptic) plasticity following DA loss leads to the emergence of abnormal self-amplified structure-function interactions within the striatum and its down-stream pathways, one can hypothesize that the progressive emergence of excessively synchronized neuronal activity in GPe or STN in PD is simultaneously accompanied (or caused) by abnormal reshaping of synaptic connectivity (also see Table 2).

### 4.1 Plasticity-Induced Changes Following Dopamine Loss

A number of experimental studies examined parkinsonian human subjects to assess the relation between DA loss and exaggerated neural oscillations and movement abnormalities (Levy et al., 2002; Hammond et al., 2007). On the other hand, by employing pharmacological agents or genetic models of DAergic neurons several experimental studies were able to mimic DA depletion in animal models to inspect the range of pathological changes that can directly or indirectly lead to the emergence of the PD condition (see Table 2). Loss of nigro-striatal DAergic neurons triggers a series of changes at multiple levels in the BG pathways which may ultimately contribute to the emergence of the PD condition. The nature of these changes, however, may be different: A number of them are compensatory mechanisms caused by homeostatic processes to counteract DA deficiency (Blesa et al., 2017), whereas others seem to be maladaptive (Villalba and Smith, 2018). Some of these changes that were experimentally observed in rats/mice slices are summarized in Table 2 based on characteristics they share:

#### 4.1. 1 Group (a)

Experimental findings on the modulatory effect of DA on the activity of striatal circuits imply that activation of DA D1Rs in striatal MSNs seems to enhance evoked activity of MSNs in response to strongly correlated glutamatergic signals from the cortex (Surmeier et al., 2007). In contrast, DA reduces glutamatergic synaptic transmission in the cortico-striatal pathway by activation of D2Rs and suppresses the response of MSNs to cortex stimulation (Surmeier et al., 2007). When DA is strongly reduced in experimental models, MSNs displayed either no significant change in their excitability or an enhanced excitability (Fino et al., 2007). This effect was attributed to the excessive glutamatergic input from cortex to striatum due to DA loss, but not the intrinsic membrane properties of MSNs (Calabresi et al., 1993). However, later it was shown that DA depletion may also increase the intrinsic excitability of MSNs (Azdad et al., 2009). The enhancement of the excitability of MSNs promotes their responsiveness to synaptic inputs which enables MSNs to relatively stabilize their firing activity after DA depletion (Azdad et al., 2009), implying that these changes are triggered by homeostatic plasticity to compensate for DA perturbations and maintain the functional range of neuronal activity in pathological conditions (Azdad et al., 2009). In addition, DA denervation alters the excitability of striatal interneurons such that the excitability of cholinergic cells is enhanced, whereas the excitability of GABAergic interneurons is suppressed (Fino et al., 2007) which can directly or indirectly determine how DA regulates MSNs.

#### 4.1.2 Group (b)

The induction of cortico-striatal LTP/LTD is bidirectional, and hence, not restricted to a particular subclass of DA receptors (Shen et al., 2008). However, given the influence of DA receptor activation on striatal MSNs (Surmeier et al., 2007) and according to observations that we summarized in Table 1, DAergic modulation of STDP occurs in a way that activation of DA D1Rs facilitates LTP of the cortico-striatal synapses, whereas D2Rs promote LTD of the cortico-striatal glutamatergic synaptic transmission (Kerr and Wickens, 2001; Pawlak and Kerr, 2008). Therefore, alternation in synaptic transmission and excitability of striatal cells in the DA-depleted condition versus the DA-intact condition affects the profile of STDP leading to undesired modification of synaptic connections. In particular, paired stimulus experiments at the cortico-striatal synapses in DA-depleted animal models failed to induce pre-post t-LTP (Centonze et al., 1999; Kerr and Wickens, 2001; Pawlak and Kerr, 2008), post-pre t-LTD (Calabresi et al., 1992b; Calabresi et al., 1997; Pawlak and Kerr, 2008) that normally follows stimulation of cortex and striatum in the presence of DA, which can impair bidirectional STDP (Picconi et al., 2003; Shen et al., 2008).

#### 4.1.3 Group (c)

The impairment of normal synaptic plasticity due to DA deficiency can lead to abnormal reshaping of structure within and between the BG nuclei. In fact, the strength of recurrent connections between MSNs in the striatum is severely down-regulated in the DA-depleted condition (Taverna et al., 2008). DA depletion leads to the elimination of glutamatergic synapses on the MSNs in the indirect pathway that can result in the disconnection of the striato-pallidal pathway from motor circuit (Day et al., 2006). In addition, deletion of DA D2 receptors from MSNs in the indirect pathway significantly increases GABAeric transmission in the striato-pallidal pathway (Lemos et al., 2016). The synaptic transmission between the GPe cells is then augmented in response to enhanced striato-pallidal transmission (Miguelez et al., 2012) leading to enhanced GABAergic synaptic transmission in the pallido-subthalamic pathway by increasing the number of bidirectional synaptic connections between GPe and STN (Fan et al., 2012), possibly due to heterosynaptic plasticity mechanisms (Chu et al., 2015). Furthermore, experimental observations suggested that cortico-subthalamic transmission may be mediated by fewer (but potentially more powerful) synapses following degeneration of midbrain DAergic neurons (Chu et al., 2017). Hence, it can be hypothesized that the resultant excitation/inhibition imbalance between the indirect and hyperdirect pathways towards the STN is pushed beyond a set point where even the homeostatic plasticity fails to restore the normal structure.

#### 4.1.4 Group (d)

Since neuronal activity and synaptic connectivity in plastic networks are tightly correlated, abnormal reshaping of structure due to impaired synaptic plasticity in DA-depleted models leads to the emergence of disrupted and pathological dynamics (neuronal activity patterns) in different BG nuclei (Moran et al., 2011; Dupuis et al., 2013). In the striatum, e.g., D1R-expressing MSNs in the parkinsonian mice fire at lower rates (0.11 ± 0.04 Hz, *n* = 14) in comparison to the control (healthy) condition (1.61 ± 0.19 Hz, *n* = 64) (Ryan et al., 2018). The results on firing rate of D2R-expressing MSNs are contradictory in some cases. Some studies reported that the mean firing rate of D2R-expressing MSNs in mice is not significantly changed in the parkinsonian condition (1.24 ± 0.23 Hz, *n* = 32) versus the control condition (1.42 ± 0.28 Hz, *n* = 34) (Ryan et al., 2018). In contrast, other experimental studies in rats reported more elevated firing rates (PD: 6.4 ± 2.7 Hz, *n* = 22 vs. control: 2.1 ± 1.2 Hz, *n* = 11) (Kita and Kita, 2011).

### 4.2 Patterned Neuronal Activity and Abnormal Synchronization

In the context of neuronal activity changes following DA loss, a widely reported experimental observation is the emergence of exaggerated beta band (15–30 Hz) oscillations in the GPe or STN (Sharott et al., 2005; Mallet et al., 2008b; Mallet et al., 2008a; Asadi et al., 2022) (for a review see (Brown et al., 2001; Hammond et al., 2007)). The conditions required for the generation of beta band oscillations in the GPe-STN network were thoroughly examined by computational studies over the past years (Bevan et al., 2002; Terman et al., 2002; Holgado et al., 2010), and therefore, we did not discuss them here. Below, we briefly mention a few experimental findings on the emergence of abnormal neuronal oscillations in GPe and STN that are relevant to group (d) in Table 2.

Changes of the firing rates of neurons in the CBGTC loop have been validated in several studies both in PD patients (Dostrovsky et al., 2000; Levy et al., 2002) and animal models (Sharott et al., 2005; Mallet et al., 2008b; Mallet et al., 2008a). It is of note that the changes of dynamics following experimental DA depletion in animals are typically characterized by altered neuronal firing patterns, relating to spiking, bursting, pausing properties or firing regularity, rather than firing rates (Johnson et al., 2009; Kita and Kita, 2011; McConnell et al., 2012; Valsky et al., 2020) and increased synchronization between neuronal populations (Sharott et al., 2005; Mallet et al., 2008b; Mallet et al., 2008a). These observations challenged the classical viewpoint according to which merely the changes in neuronal firing rates underlie PD motor symptoms and suggested that changes in neuronal firing patterns may be crucial (Kita and Kita, 2011; McConnell et al., 2012).

During cortical activation, the mean firing rate of the GPe neurons is suppressed in parkinsonian rats (14.6 ± 0.4 Hz, *n* = 149) compared with the corresponding control condition (33.7 ± 1.3 Hz, *n* = 478) (Mallet et al., 2008a). Notably, 44.2% of the GPe neurons in the PD condition were synchronized at beta frequency firing compared with 0.6% of neurons in the control condition (Mallet et al., 2008a). Spontaneous activity pattern of the GPe neurons also showed a relatively suppressed firing rate in parkinsonian rats (26.2 ± 10.2 Hz, *n* = 54) versus the control condition (29.3 ± 12.2 Hz, *n* = 52) (Kita and Kita, 2011). In this case, both the bursting index (fraction of spikes in bursts over a total number of spikes) and pausing fraction (fraction of total time occupied by pauses), as well as the number of pauses in the activity of the GPe cells were significantly increased in the PD condition (bursting index: 44.3 ± 15.6%; pausing fraction: 23.2 ± 5.2%; number of pauses/min: 55.6 ± 14.8%) compared to the control condition (bursting index: 13.1 ± 14.2%; pausing fraction: 11.9 ± 4.1%; number of pauses/min: 26.8 ± 17.3%) (Kita and Kita, 2011).

It has been suggested that motor cortex input to the STN regulates synaptic transmission in the pallido-subthalamic pathway via heterosynaptic plasticity mechanisms (Chu et al., 2015). In the normal condition, this heterosynaptic plasticity is homeostatic which balances excitatory inputs from the hyperdirect pathway and inhibitory inputs from the indirect pathway by adjusting the number of functional synaptic connections. Following DA depletion, heterosynaptic plasticity abnormally strengthens pallido-subthalamic synaptic connections (Chu et al., 2015), whereas the number of cortico-subthalamic synapses is decreased (but not necessarily the strength of inputs) (Chu et al., 2017). This ultimately disturbs excitatory-inhibitory input balance towards the STN and could underlie the emergence of abnormally synchronized oscillatory activity at beta frequencies (15–30 Hz) in the PD condition. The emergence of abnormal beta band neuronal oscillations in the STN is a widely reported observation in experimental PD models (Sharott et al., 2005; Mallet et al., 2008b). For instance, it has been shown that the mean firing rate of the STN cells in rats slices is significantly enhanced (34.0 ± 3.4 Hz, *n* = 9) in comparison to the control condition (13.8 ± 2.7 Hz, *n* = 8) during activated brain state (Mallet et al., 2008b).

Several computational studies focused on how synchronization and desynchronization properties of neurons are related to the their firing patterns. For instance, bursting neurons can show stable synchronous states of firing depending on network topology and coupling strengths (Belykh et al., 2005). Spiking neuronal populations can generate network-level oscillations despite an irregular (i.e., non-oscillatory) discharge pattern of single neurons and a low firing rate (Brunel, 2000). By using a simple spiking neuron model, Brunel and colleagues showed that simple networks can exhibit different modes of neuronal firing ranging from synchronous states with regular firing of single neurons to asynchronous states with stationary global activity and irregular single cell activity (Brunel, 2000). By changing the excitation-inhibition balance, the network dynamics can switch between these states (Brunel, 2000). In fact, synaptic plasticity may play a key role in controlling the transitions between different neuronal activity modes.

### 4.3 Multistability and Activity-Connectivity Coevolution Mediated by STDP

The connectivity pattern in plastic networks is modified by STDP according to the neuronal activity. In return, the synaptic connectivity can determine the neuronal activity in a feedback cycle. In this way, STDP leads to the coevolution of activity-connectivity patterns (Madadi Asl et al., 2017; Madadi Asl et al., 2018c). In fact, transitions between synchronized and desynchronized neuronal firing states can be facilitated by assuming synaptic plasticity in network models with adaptive synapses that can further stabilize network dynamics in either one of these states (Lubenov and Siapas, 2008; Berner et al., 2021). STDP in simple regular spiking neuron models can stabilize the network activity in both synchronizing and desynchronizing states by rewiring of the synaptic connections between neurons depending on the range of the transmission delays between neurons (Lubenov and Siapas, 2008). The presence of opposing synchronizing and desynchronizing forces due to STDP may promote self-organization of network activity giving rise to mixture states at the border between randomness and synchronous bursts (Lubenov and Siapas, 2008).

**FIGURE 3:**
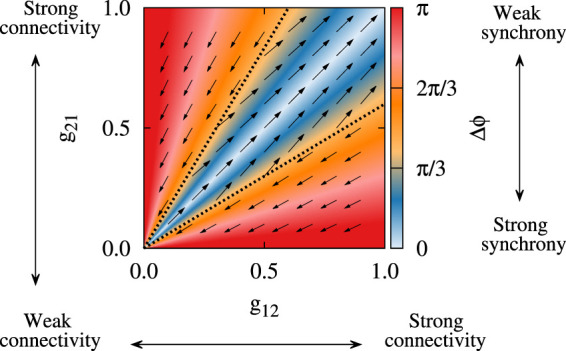
Bistability in a two-neuron system with plastic synapses subjected to STDP. The colors show the stable fixed point of the phase lag (Δ*ϕ*) between the spiking of the neurons and the vector field shows the direction of the change of the plastic synaptic strengths (*g*_21_, *g*_12_). Depending on the initial synaptic coupling, bistable attractor states may finally emerge due to STDP, i.e., strongly synchronized states with strong synaptic connections (blue region) as opposed to desynchronized states with weaker synaptic connections (red region). Adapted from Madadi Asl et al. (2018a) with no permission required.

As shown computationally, STDP can induce multistable dynamics in neuronal networks, i.e., qualitatively different stable states may emerge such as strongly synchronized states with strong synaptic connections as opposed to desynchronized states with weaker synaptic connections (Tass and Majtanik, 2006; Maistrenko et al., 2007; Popovych and Tass, 2012; Madadi Asl et al., 2018a). On the other hand, experimental parkinsonism is characterized by pathological neuronal synchronization and synaptic connectivity within and between several nuclei (Sharott et al., 2005; Mallet et al., 2008b; Miguelez et al., 2012; Fan et al., 2012) as opposed to the physiological condition. Computationally, this relates to a bistability or multistability between pathological and physiological states, i.e., strongly synchronized states with strong synaptic connections (pathological states) as opposed to desynchronized states with weaker synaptic connections (physiological states), as illustrated in Figure 3 by modeling neurons with plastic synapses subjected to STDP. Moreover, multistability may not only occur on a collective neuronal level, but also on a single-neuron level, as revealed by the study of firing properties of STN neurons in PD patients (Chopek et al., 2019).

Computationally, the presence of long-term activity-dependent synaptic changes in the BG subnetworks may provide a substrate for STDP to favorably/unfavorably regulate emergent patterns of neuronal activity and synaptic connectivity (Ebert et al., 2014; Lourens et al., 2015). For instance, a computational model of DA-mediated STDP addressed the distal reward problem by describing how precise firing patterns of neurons can influence synaptic connections through synaptic plasticity (Izhikevich, 2007). STDP-induced abnormal changes in the BG may be one of the mechanism behind the gradual appearance of PD symptoms since activity-connectivity interactions fail to function normally due to DA loss. Hence, it can be hypothesized that DAergic modulation of plasticity mechanisms is critical for stabilization of healthy basins of attraction of neuronal activity, i.e., stable modes of neuronal activity, in the BG (Madadi Asl et al., 2019), where alterations in DA signaling can lead to the long-term reshaping of inter-population synaptic connectivity (Fan et al., 2012; Chu et al., 2017). Furthermore, the capability of STDP to rewiring the synaptic connections simultaneously reshapes the structure and dynamics of neuronal networks and can be employed to calibrate therapeutic brain stimulation techniques aimed at the treatment of PD by destabilizing the diseased basins of attraction (Tass and Majtanik, 2006; Lourens et al., 2015; Kromer and Tass, 2020; Schwab et al., 2021).

## 5 Conclusion

Dysfunction of the BG circuitry is implicated in movement disorders. For instance, PD is associated with multi-systemic neurodegeneration in the BG (Surmeier et al., 2017; McGregor and Nelson, 2019). Several mechanisms contribute to the progress of the neurodegeneration. One of the most common indicators in PD pathogenesis is the presence of Lewy bodies (comprising *α*-synuclein protein). According to the leading hypothesis the pathological fibrils spread through the connectome in a primarily retrograde manner (Wakabayashi et al., 2007), which is often accompanied with neuronal degeneration (Surmeier et al., 2017). However, build-up of Lewy bodies and neurodegeneration can be notably detected in the midbrain and DAergic neurons embedded in the SNc of PD patients (McGregor and Nelson, 2019). Some PD symptoms are linked to widespread neuronal loss, other symptoms are linked to abnormal neuronal activity (McGregor and Nelson, 2019). Particularly, motor symptoms of PD are linked to significant degeneration of DAergic neurons and their projections to the striatum which is followed by BG circuit dysfunction (Brown et al., 2001; Mallet et al., 2008b). Apart from a loss of DAergic neurons, there is also a loss of serotonergic and noradrenergic neurons that can contribute to the emergence of pathological structural and dynamical changes in PD condition (McGregor and Nelson, 2019).

However, neurodegeneration may not simply cause the emergence of PD-related activity in a causally unidirectional manner. Abnormal neuronal activity and synaptic connectivity may rather fundamentally contribute to and facilitate ongoing neurodegeneration in PD. By simultaneous modulation of neuronal activity and synaptic connectivity, STDP shapes the dynamics and structure in BG networks. We provided a review of STDP-related experimental studies addressing the modulatory effect of DA signaling on synaptic plasticity. The discussed experimental studies enable an understanding of the possible role of impaired synaptic plasticity and, especially, STDP in the emergence of PD symptoms due to the cascade of maladaptive and compensatory changes within the BG following DA loss. However, this remains to be studied in detail by future experimental studies to reveal the exact DA-mediated plasticity mechanisms within and between different BG nuclei and to reveal their role in shaping abnormal structure-function relationship in PD.

Synaptic plasticity and its DA-induced changes are the target of dedicated invasive and non-invasive stimulation therapies, such as coordinated reset (CR) deep brain stimulation (DBS) (Tass et al., 2012; Adamchic et al., 2014; Wang et al., 2016) and vibrotactile CR stimulation (Tass, 2017; Syrkin-Nikolau et al., 2018; Pfeifer et al., 2021; Tass, 2022). Better understanding of dopamine-related alterations of synaptic plasticity may enable further improvements of therapies aiming at a reversal of abnormal synaptic connectivity in order to restore physiological function.

## Abbreviations

6-OHDA, 6-hydroxydopamine; BCM, Bienenstock-Cooper-Munro; BG, basal ganglia; CBGTC, cortico-basal ganglia-thalamocortical; CR, coordinated reset; DA, dopamine; DAergic, dopaminergic; DBS, deep brain stimulation; EPSC, excitatory postsynaptic current; EPSP, excitatory postsynaptic potential; GABA, gamma-aminobutyric acid; GPe, external globus pallidus; GPi, internal globus pallidus; HFS, high-frequency stimulation; IPSP, inhibitory postsynaptic potential; L-DOPA, l-3,4-dihydroxyphenylalanine; LFS, low-frequency stimulation; LTD, long-term depression; LTP, long-term potentiation; MPTP, 1-methyl-4-phenyl-1,2,3,6-tetrahydropyridine; MSN, medium spiny neuron; NAc, nucleus accumbens; PD, Parkinson’s disease; SNc, substantia nigra pars compacta; SNr, substantia nigra pars reticulata; STDP, spike-timing-dependent plasticity; STN, subthalamic nucleus; t-LTD, timing-dependent long-term depression; t-LTP, timing-dependent long-term potentiation; VTA, ventral tegmental area.
